# Insomnia and its correlates in a representative sample of the Greek population

**DOI:** 10.1186/1471-2458-10-531

**Published:** 2010-09-03

**Authors:** Thomas Paparrigopoulos, Chara Tzavara, Christos Theleritis, Constantin Psarros, Constantin Soldatos, Yiannis Tountas

**Affiliations:** 1 University of Athens Medical School, 1st Department of Psychiatry, Eginition Hospital, Athens, Greece; 2 University of Athens Medical School, Center for Health Services Research, Athens, Greece

## Abstract

**Background:**

Insomnia is a major public health concern affecting about 10% of the general population in its chronic form. Furthermore, epidemiological surveys demonstrate that poor sleep and sleep dissatisfaction are even more frequent problems (10-48%) in the community. This is the first report on the prevalence of insomnia in Greece, a southeastern European country which differs in several socio-cultural and climatic aspects from the rest of European Community members. Data obtained from a national household survey (n = 1005) were used to assess the relationship between insomnia symptoms and a variety of sociodemographic variables, life habits, and health-related factors.

**Methods:**

A self-administered questionnaire with questions pertaining to general health and related issues was given to the participants. The Short Form-36 (Mental Health subscale), the Athens Insomnia Scale (AIS) as a measure of insomnia-related symptoms, and the International Physical Activity Questionnaire (IPAQ) were also used for the assessment.

**Results:**

The prevalence of insomnia in the total sample was 25.3% (n = 254); insomnia was more frequent in women than men (30.7% vs. 19.5%) and increased with age. Multiple regression analysis revealed a significant association of insomnia with low socio-economical status and educational level, physical inactivity, existence of a chronic physical or mental disease and increased number of hospitalizations in the previous year.

**Conclusions:**

The present study confirms most findings reported from other developed countries around the world regarding the high prevalence of insomnia problems in the general population and their association with several sociodemographic and health-related predisposing factors. These results further indicate the need for more active interventions on the part of physicians who should suspect and specifically ask about such symptoms.

## Background

Insomnia is a major public health concern affecting about 10% of the general population in its chronic form. Moreover, epidemiological surveys demonstrate that poor sleep and sleep dissatisfaction are even more frequent problems in the community, ranging from 10-48%, depending on the study [1-9]. Several correlates of insomnia have been identified, such as sociodemographic determinants, life habits, mental disorders and physical illnesses [1]. It is generally acknowledged that women are more likely than men to report insomnia symptoms, daytime consequences of disturbed sleep and sleep dissatisfaction, and consequently to receive a diagnosis of insomnia [3-5,10-18]. Thus, women/men ratio for insomnia symptoms is approximately 1.4; this ratio increases with age, reaching 1.7 after 45 years of age [1]. Also, the vast majority of epidemiological studies report an increased prevalence of insomnia symptoms and sleep dissatisfaction with age, approaching 50% in the elderly population [4,5,13-15,17,19,20], when it reaches a plateau, women suffering more often than men from such symptoms [1-5,21,22]. In regard to other sociodemographic determinants most studies report a higher prevalence of insomnia in separated, divorced, or widowed individuals [4,5,16,17,23], women in particular [4,5], in less privileged individuals of lower income [4,19], of lower education [19,20,24] or unemployed [4,5,14,15,23]; the highest risk of insomnia symptomatology has been found in retired people, followed by housekeepers [5].

Regarding the various factors connected with insomnia these have been divided into primary, secondary (mental disorders, medical conditions, sleep disorders) and self-induced factors (lifestyle, use, abuse or withdrawal of psychoactive substance) [1]. For what concerns mental health disorders in particular, insomnia symptoms may be present in the large majority (over 80%) of individuals suffering from major depression and in more than one third of cases with any mental disorder [1,25-30]. Furthermore, persistent insomnia symptoms may increase the likelihood of developing major depressive disorder [1,31] and may be a risk factor of physical health problems as well [1,32].

This is the first report on the prevalence of insomnia in Greece, a southeastern European country which differs in several socio-cultural and climatic aspects from the rest of European Community members. Thus, Greece has a warmer climate than the rest of Europe and daylight hours are relatively extended compared to other latitudes, which gives more opportunities for social evening activities and leads to delayed bedtime hours. Furthermore, in the Greek culture daytime napping remains a socially acceptable behavior, even in large cities; although a continuous working schedule has been operating in most cases during the last two decades, napping behavior still appears to be relatively prevalent in Greece. Finally, because strong emotional and financial bonds still exist within both the core and extended Greek family, housing conditions of the family may differ from those in the other European countries. The present study also investigates the relationship between insomnia and a variety of sociodemographic parameters, life habit factors and health factors in a representative sample of the Greek population.

## Methods

### Sample

Data were obtained from the national household survey Hellas Health I, conducted during 2006 by the Center of Health Services Research of the Department of Hygiene and Epidemiology, Medical School, University of Athens. Candidate respondents were selected by means of a three-stage, proportional-to-size sampling design. First, a random sample of building blocks was selected proportionally to size. Second, in each selected area of blocks, the households to be interviewed were randomly selected by means of systematic sampling. Third, in each household, a sample of individuals aged 18 years or above was selected by means of simple random sampling. The survey covered urban areas (2000 or more inhabitants) and rural areas all over Greece. Patients in hospitals, sheltered homes, and homeless people were not investigated.

The survey population consisted of 1005 individuals and effective response rate reached 44.5%, which is a fairly good rate for Greek standards. All participants were interviewed face to face by trained interviewers. The duration of the interview was 35-40 minutes. The sample was representative of the Greek population in terms of age and residence. Approval for the study was obtained from the institutional review board of the Athens University Medical School, and the protocol conformed to the ethical guidelines of the 1975 Declaration of Helsinki.

### Assessments

A self-administered questionnaire including 146 questions or sub-questions and 13 open-ended questions pertaining to general health and related issues was given to the participants; the questionnaire had been pre-tested in terms of the comprehension and the order of the questions included. The socio-demographic questions were close-ended and included age, sex, marital status, residency (urban vs. rural), educational level and social class. Social class was based on the ESOMAR 1997 index categories (i.e., calculated on the basis of the family's main income earner's job category and the level of education and summed up into three social categories: A/B-C1 = upper/upper middle, C2 = lower middle and D/E = lower).

Self reported measures of weight (kg) and height (m) were obtained by the questionnaire. Body mass index (BMI) was calculated as weight/(height)^2^. Respondents were also asked to report any chronic diseases from a checklist of the leading causes of morbidity (i.e., diabetes, hypertension, hypercholesterolemia or other chronic diseases); the number of chronic diseases of the respondents was evaluated. Current smokers were defined as those who smoked at least one cigarette per day. Alcohol use was defined as at least one drink per day. Physical activity was evaluated using the International Physical Activity Questionnaire (IPAQ) [33]. The IPAQ was graded in qualitative terms as sedentary (score on IPAQ less than 30), light (score on IPAQ from 30 to 41.5), moderate (score on IPAQ from 42 to 59.5) and vigorous (score on IPAQ more than 60). Subjects with score on IPAQ less than 30 were characterized as physically inactive.

Insomnia symptoms were assessed through the Athens Insomnia Scale (AIS), which is an 8-item standardized self-assessment psychometric instrument designed for quantifying sleep difficulty based on the ICD-10 criteria. It consists of eight items: the first five pertain to sleep induction, awakenings during the night, final awakening, total sleep duration, and sleep quality; while the last three refer to well-being, functioning capacity, and sleepiness during the day [34]. A cut-off score of ≥6 on the AIS was used to establish the diagnosis of insomnia [35]. Mental health was assessed using the 'mental health summary score' from the Short Form-36 self-administered questionnaire [36] (SF-36, Greek standard version 1.0).

### Variables

Insomnia was evaluated in association with the following variables:

1. Sociodemographic factors: age, sex, marital status, socioeconomical status and educational level.

2. Life habit factors: use of alcohol, tobacco and physical activity level.

3. Health factors: chronic disease, mental health, body mass index and the number of hospitalizations in the previous year.

### Statistical analysis

Quantitative variables are presented with absolute and relative frequencies. For comparisons between proportions chi-square test was used. P_value for trend in the prevalence of insomnia by age was also calculated. Univariate logistic regression analyses were used to test the effect of the factors under investigation on having insomnia and data were modeled using logistic regression analysis. Odds ratios (OR) with 95% confidence intervals (95% CI) were computed from the results of the logistic regression analyses. All p values reported are two-tailed; statistical significance was set at 0.05. Analyses were conducted using the SPSS statistical software (version 13.0).

## Results

**Figure 1:**
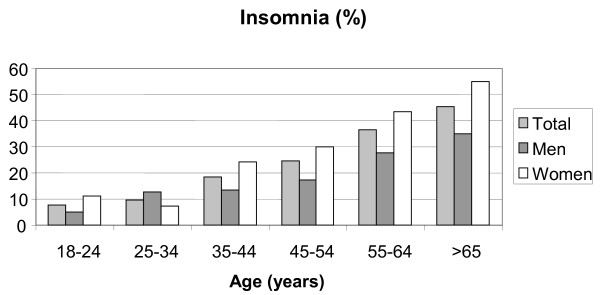
**The prevalence of insomnia presented by sex and age groups**.

Data from 1005 participants were analyzed. Sample characteristics are presented in Table 1. The prevalence of insomnia for the total sample was 25.3% (95% Confidence Interval: 22.6% - 28.0%). Insomnia was more frequent in women than men (30.7% vs. 19.5%, p < 0.001) and increased by age (p for trend <0.001 - Figure 1). Regarding the specific sleep complaints as reflected in the AIS, these were mainly related to delayed sleep onset and increased number of awakenings during the night, both for men and women. Of the total sample, 29.2% had at least one of the items of AIS-8 rated as 'markedly' or 'severely impaired'; 11.5% reported that their final awakening was at least 'markedly earlier' than desired, while 9.9% estimated that their total sleep time was at least 'markedly unsatisfactory'; 10.2% of the total sample (7.9% of men and 12.5% of women) complained for their sleep quality. Complaints regarding well-being, functioning and sleepiness during the day were made by 8.6%, 8.6% and 5.7% of the respondents (Table 2).

**Table 1:** Sample characteristics

	N (%)
Sex	
Men	483 (48.1)
Women	522 (51.9)
Age (years)	
18-24	115 (11.4)
25-34	185 (18.4)
35-44	180 (17.9)
45-54	151 (15.0)
55-64	150 (14.9)
> 65	224 (22.3)
Family status	
Married	646 (64.3)
Single	244 (24.3)
Divorced/Widowed	115 (11.4)
Socioeconomic status*	
Upper/upper middle (A/B-C1)	168 (16.7)
Lower middle (C2)	462 (46.0)
Lower (D/E)	375 (37.3)
Level of education	
Low	334 (33.2)
Middle	422 (42.0)
High	249 (24.8)
Alcohol use	
No	453 (45.1)
Yes	552 (54.9)
Smoking	
No	572 (56.9)
Yes	433 (43.1)
Physical activity	
No	478 (50.5)
Yes	469 (49.5)
Weight status	
Normal (BMI < 25)	416 (42.4)
Overweight (25<BMI < 30)	404 (41.2)
Obese (BMI > 30)	161 (16.4)
Chronic disease	
No	645 (64.2)
Yes	360 (35.8)
Hospitalizations in the past 12 months	
None	880 (88.4)
One or more	115 (11.6)
Mental health problems	
Present	50 (5.0)
Absent	955 (95.0)
SF-36 Mental health Component Score	
Individuals with mental problems	37.6 (SD = 11.0)
Individuals without mental problems	48.1 (SD = 8.9)

*based on the ESOMAR 1997 index categories

**Table 2:** Prevalence of sleep complaints (based on AIS) in the total sample and by gender. Number of responders (%) who estimate to have a 'marked' or 'severe problem' in the eight AIS items

	Total sample	Men	Women
*AIS-8 individual items*	N (%)	N (%)	N (%)
Sleep induction	174 (17.3)	50 (10.4)	124 (23.8)
Awakenings during the night	147 (14.6)	50 (10.4)	97 (18.6)
Final awakening	116 (11.5)	46 (9.5)	70 (13.4)
Total sleep duration	99 (9.9)	34 (7.0)	65 (12.5)
Sleep quality	103 (10.2)	38 (7.9)	65 (12.5)
Well-being during the day	86 (8.6)	35 (7.2)	51 (9.8)
Functioning during the day	86 (8.6)	28 (5.8)	58 (11.1)
Sleepiness during the day	57 (5.7)	19 (3.9)	38 (7.3)

In univariate analysis odds for insomnia was lower for single subjects and higher for divorced/widowed subjects compared to married individuals but this association was not significant in the multivariate analysis (Table 3). Participants of low socioeconomical status suffered more often from insomnia than those of high socioeconomical status; this association remained significant after adjusting for other variables (OR = 1.67, 95% CI: 1.03-2.69). The difference in the odds for insomnia was not significant between individuals of low and individuals of middle socioeconomical status. The educational level was a significant predictor for insomnia both in univariate and multivariate analysis. Thus, individuals of middle or high educational level had a lower likelihood for having insomnia compared to those of low educational level. Alcohol and smoking were significant predictors in the bivariate analysis but did not make an independent contribution to insomnia in the multivariate analysis. On the other hand, physical activity was a significant predictor for insomnia. Physically inactive subjects had 1.42 greater odds for having insomnia in the multivariate model. Obese subjects had a higher prevalence of insomnia than subjects of normal weight but this association was not significant after adjusting for other variables. Finally, chronic disease, the number of hospitalizations in the previous year and mental health significantly predicted insomnia in the multiple logistic regression analysis with odds ratios 1.58 (95% CI: 1.06-2.34), 1.90 (95% CI: 1.18-3.05) and 0.96 (95% CI: 0.95-0.97) respectively.

**Table 3:** Prevalence of insomnia and associations derived by logistic regression analysis

Variable	Insomnia, N (%)	OR (95% CI) Crude	OR (95% CI) Adjusted
Sex			
Men	94(19.5)	1.00	1.00
Women	160(30.7)	1.83* (1.37-2.45)	1.48‡ (1.03-2.10)
Age (years)			
18-24	9(7.8)	1.00	1.00
25-34	18(9.7)	1.26(0.55-2.93)	1.16(0.48-2.81)
35-44	33(18.3)	2.64‡ (1.21-5.75)	2.09(0.90-4.83)
45-54	37(24.5)	3.82*(1.76-8.29)	2.68‡ (1.17-6.14)
55-64	55(36.7)	6.82*(3.19-14.54)	3.63† (1.59-8.28)
> 65	102(45.5)	9.84*(4.74-20.42)	4.06† (1.79-9.17)
Family status			
Married	179(27.7)	1.00	1.00
Single	23(9.4)	0.27* (0.17-0.43)	0.65(0.36-1.17)
Divorced/Widowed	52(45.2)	2.15* (1.44-3.23)	1.04(0.62-1.74)
Socioeconomic status			
A/B-C1	30(17.9)	1.00	
C2	116(25.1)	1.54(0.99-2.41)	1.40(0.88-2.24)
D/E	108(28.8)	1.86†(1.18-2.93)	1.67‡ (1.03-2.69)
Level of education			
Low	140(41.9)	1.00	1.00
Middle	73(17.3)	0.29* (0.21-0.40)	0.59‡ (0.38-0.91)
High	41(16.5)	0.27* (0.18-0.41)	0.58‡ (0.36-0.94)
Alcohol use			
No	154(34.0)	1.00	1.00
Yes	100(18.1)	0.43* (0.32-0.58)	0.56 (0.41-1.19)
Smoking			
No	165(28.8)	1.00	1.00
Yes	89(20.6)	0.64† (0.48-0.86)	0.96(0.65-1.41)
Physical activity			
No	84(17.9)	1.00	1.00
Yes	153(32.0)	2.16* (1.59-2.92)	1.42‡ (1.01-2.03)
Weight status			
Normal (BMI < 25)	86(20.7)	1.00	1.00
Overweight (25≤BMI < 30)	100(24.8)	1.26(0.92-1.75)	0.78(0.52-1.16)
Obese (BMI≥30)	60(37.3)	2.28* (1.53-3.39)	1.06(0.66-1.72)
Chronic disease			
No	100(15.5)	1.00	1.00
Yes	154(42.8)	4.07* (3.02-5.49)	1.58‡ (1.06-2.34)
Hospitalizations in the past 12 months			
None	201(22.8)	1.00	1.00
One or more	50(43.5)	2.60* (1.74-3.88)	1.90‡ (1.18-3.05)
Mental health summary score (SF-36)		0.95*(0.94-0.96)	0.96*(0.95-0.97)

OR = Odds Ratio, CI = 95% Confidence Interval, ‡ p < 0.05, † p < 0.01, * p < 0.001

## Discussion

This is the first epidemiological study to evaluate the prevalence of insomnia problems and its correlates in the general population of Greece. Insomnia, as measured by the Athens Insomnia Scale (AIS), was reported by 25.3% of the sample; prevalence of insomnia was significantly higher in women than in men (30.7% vs. 19.5%) and considerably increased with age. These findings are in accordance with the existing literature, although estimates of the prevalence of insomnia may widely vary depending on the applied definition of insomnia and the methodology used [1]. In a large-scale single-day survey in ten countries across the globe where the Athens Insomnia Scale was used as a self-assessment instrument, 31.6% of the subjects reported to have 'insomnia' while another 17.5% could be considered as having 'sub-threshold insomnia'; however, in this study important global variations in the prevalence of insomnia were observed [37].

Marital and socioeconomic status is considered to be of importance among the sociodemographic determinants of insomnia. This was also replicated in the present study. Thus, participants of lower socioeconomic and educational level were more likely to suffer from insomnia even after correction for possible confounding factors such as sex and age. Furthermore, although in univariate analysis the odds for insomnia was lower for single subjects but higher for divorced or widowed subjects compared to married individuals, this association was not significant in multivariate analysis. Consequently, marital status did not contribute in a significant way to the occurrence of insomnia contrary to what is generally reported in the literature, although this correlation may be more prominent in the female population [4,5,16,17,23].

Concerning life habits, which may affect sleep quality and lead to non restorative sleep complaints [38], alcohol and smoking were significant predictors for insomnia in bivariate analysis but did not make an independent contribution in the multivariate analysis. Clearly, more detailed information on such habits is necessary in order to investigate their potential influence on sleep, which was not provided by the study. On the other hand, physical activity was a significant predictor for insomnia, i.e., physically inactive subjects were more at risk for having insomnia compared to active individuals. Epidemiological surveys have demonstrated sleep promoting benefits of moderate, regular physical activity although experimental evidence from sleep studies is not so compelling [39]; however, in a recent study based on the same study population it has been shown that physical activity may promote sleep in cardiac patients suffering from insomnia [40].

Obesity was another predictor for insomnia but the association was not significant after adjusting for other variables. This is in line with the findings of another large epidemiological study where it has been reported that body mass index (BMI) was not significantly related to non restorative sleep complaints [38]. This was somewhat unexpected because overweight people frequently suffer from a variety of physical illnesses as well as sleep apnea syndromes, which may have a considerable impact on sleep; a more meticulous investigation of this factor is therefore warranted in future epidemiological studies. Regarding the other health factors investigated, i.e., the presence of a chronic disease and mental health problems, these significantly predicted insomnia in the multiple regression analysis; this finding is in accordance with the existing literature [1,29,30,41].

## Conclusions

There are some inherent limitations to this epidemiological study. First, prevalence of insomnia was not based on objective measures, i.e., polysomnography or actigraphy data. Second, because the study was part of a general health epidemiological survey, no detailed data related to specific sleep disorders or sleep aids and medications use were collected; finally, circadian sleep-wake parameters, such as shift work, were not assessed. Despite the above limitations, the present study confirms most findings reported from other developed countries around the world, that is, the high prevalence of insomnia problems in the general population (25.3%) and their association with female sex, older age, a low socio-economic-educational level, physical inactivity, and the existence of a chronic physical or mental disease in the previous year. These findings further indicate that insomnia symptoms is a major public health issue, as well as the need for more active interventions especially on the part of mental health professionals who should suspect and specifically ask about such symptoms, since only a modest percentage (27-45%) of insomnia complainers will discuss such problems with their physicians [1-9].

## Competing interests

The authors declare that they have no competing interests.

## Authors' contributions

ThP, ChTz, ChTh, and CP participated in the preparation of the paper. CS and YT had overall supervision of the study. All authors read and approved the final manuscript.

## Pre-publication history

The pre-publication history for this paper can be accessed here:

http://www.biomedcentral.com/1471-2458/10/531/prepub
